# Radiation induces premature chromatid separation via the miR-142-3p/Bod1 pathway in carcinoma cells

**DOI:** 10.18632/oncotarget.11080

**Published:** 2016-08-05

**Authors:** Dong Pan, Yarong Du, Zhenxin Ren, Yaxiong Chen, Xiaoman Li, Jufang Wang, Burong Hu

**Affiliations:** ^1^ Key Laboratory of Heavy Ion Radiation Biology and Medicine of Chinese Academy of Sciences & Key Laboratory of Space Radiobiology of Gansu Province, Institute of Modern Physics, Chinese Academy of Sciences, Lanzhou, China; ^2^ University of Chinese Academy of Sciences, Beijing, China; ^3^ Collaborative Innovation Center of Radiation Medicine of Jiangsu Higher Education Institutions and School for Radiological and Interdisciplinary Sciences (RAD-X), Soochow University, Suzhou, Jiangsu, China

**Keywords:** radiation, premature chromatid separation, miR-142-3p, Bod1, radiosensitivity

## Abstract

Radiation-induced genomic instability plays a vital role in carcinogenesis. Bod1 is required for proper chromosome biorientation, and Bod1 depletion increases premature chromatid separation. MiR-142-3p influences cell cycle progression and inhibits proliferation and invasion in cervical carcinoma cells. We found that radiation induced premature chromatid separation and altered miR-142-3p and Bod1 expression in 786-O and A549 cells. Overexpression of miR-142-3p increased premature chromatid separation and G2/M cell cycle arrest in 786-O cells by suppressing Bod1 expression. We also found that either overexpression of miR-142-3p or knockdown of Bod1 sensitized 786-O and A549 cells to X-ray radiation. Overexpression of Bod1 inhibited radiation- and miR-142-3p-induced premature chromatid separation and increased resistance to radiation in 786-O and A549 cells. Taken together, these results suggest that radiation alters miR-142-3p and Bod1 expression in carcinoma cells, and thus contributes to early stages of radiation-induced genomic instability. Combining ionizing radiation with epigenetic regulation may help improve cancer therapies.

## INTRODUCTION

Radiation-induced genomic instability (RIGI), a common long-term complication resulting from exposure to ionizing radiation [1], is characterized by an increase in genomic alterations in the progeny of irradiated cells that are associated with karyotypic abnormalities, gene mutation and amplification, cellular transformation, clonal heterogeneity, and delayed reproductive cell death [2]. Currently, the mechanisms underlying RIGI are unknown [3–7], although genomic instability plays a crucial role in radiation-induced carcinogenesis. Therefore, investigations of RIGI may identify important processes that promote radiation-induced carcinogenesis [8] and improve radiotherapy treatments for cancer patients.

Biorientation of chromosomes in cell division 1 (Bod1), a novel protein found in vertebrate centrosomes and outer kinetochores, locates and corrects aberrant syntelic attachments in mitotic spindles and is required for proper chromosome biorientation [9]. Bod1 depletion decreases sister chromatid cohesion and increases premature chromatid separation [10]. Because energy deposited by ionizing radiation induces DNA damage by breaking, rearranging, and otherwise altering chromosomes [8], Bod1 might play an important role in cells during irradiation.

MicroRNAs (miRNAs) are an important class of conserved, small noncoding RNAs that target the 3′-UTRs of target mRNAs to repress mRNA expression post-transcriptionally or promote mRNA degradation [11–14]. miRNAs play crucial regulatory roles in a wide range of biological processes, including proliferation, differentiation, apoptosis, and cell mobility [15, 16], as well as carcinogenesis, in which they alter the expression of oncogenes and tumor suppressor genes [17]. For example, miR-142-3p, which acts as a tumor suppressor, is down-regulated in many different cancers [18–20]. Tang *et al.* recently found that miR-142-3p expression was lower in cervical carcinoma cells than in normal cervical epithelium cells [21], and Deng *et al.* reported that miR-142-3p inhibits cervical cancer cell proliferation and invasion by targeting frizzled class receptor 7 (FZD7) [14]. MiR-142-3p also inhibits cancer cell proliferation and induces cell cycle arrest in the G2/M phase by targeting CDC25C [22]. However, the biological functions of miR-142-3p remain largely unknown, especially with regard to cellular radiation responses. Bioinformatics predictions (Target Scan and microRNA.org) suggest that miR-142-3p targets the Bod1 gene. Whether miR-142-3p expression is altered by irradiation, and whether it targets Bod1 to induce chromosomal aberrations after irradiation, remains unknown.

In this study, we found that radiation induced premature chromatid separation in 786-O and A549 cells. In addition, irradiation altered the expression of both miR-142-3p and Bod1. MiR-142-3p targeted the Bod1 3′-UTR sequence and inhibited its expression, and overexpression of miR-142-3p induced premature chromatid separation and G2/M arrest in 786-O cells by inhibiting Bod1. Furthermore, either overexpression of miR-142-3p or knockdown of Bod1 sensitized 786-O and A549 cells to X-ray radiation.

## RESULTS

### Radiation induces premature chromatid separation in 786-O and A549 cells

**Figure 1:**
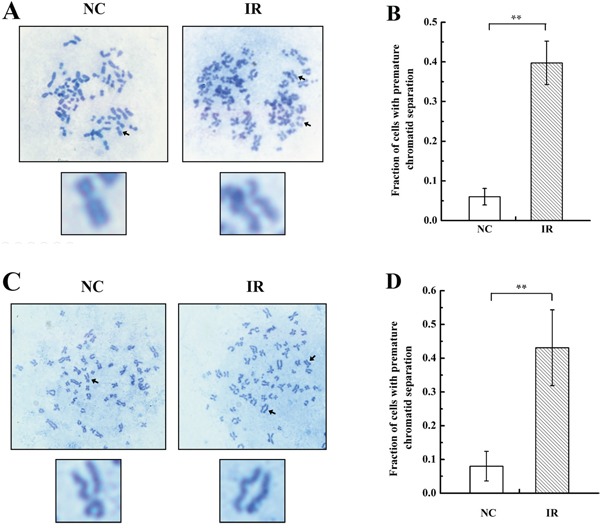
Radiation induces premature chromatid separation in 786-O and A549 cells **A** & **C.** Metaphase spreads from 786-O and A549 cells after 4 Gy X-ray irradiation (IR) or negative control (NC) treatment. Arrows in the blown-up images indicate a normal chromosome in an NC cell and premature separation of sister chromatids in an IR cell. **B** & **D.** Histogram of the proportions of IR and NC 786-O and A549 cells with premature chromatid separation based on chromosome configuration analysis. Each data point represents the mean of three separate experiments; bars indicate standard errors. **P < 0.01.

RIGI promotes the acquisition of genetic alterations, including karyotypic abnormalities [3, 4], of which premature chromatid separation is one type [23]. We therefore measured premature chromatid separation in irradiated and un-irradiated cells by analyzing chromosome configurations (Figure 1A and 1C) in 786-O and A549 cells 24 h after 4Gy X-ray irradiation. As shown in Figure 1, radiation increased premature chromatid separation in both 786-O (Figure 1B) and A549 cells (Figure 1D) compared to un-irradiated cells.

### Irradiation alters miR-142-3p and Bod1 expression in 786-O cells

**Figure 2:**
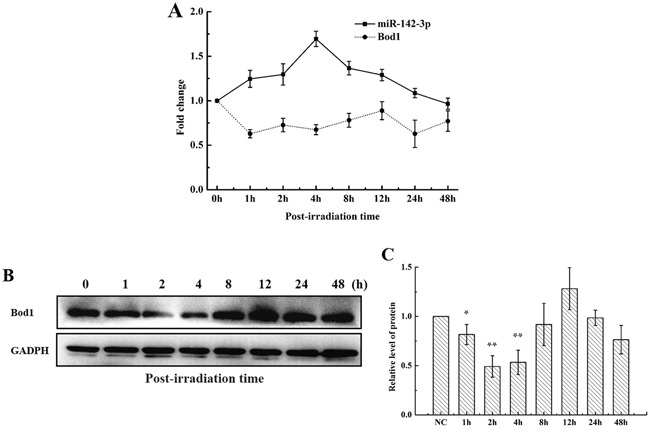
Radiation alters miR-142-3p and Bod1 levels **A.** Relative miR-142-3p and Bod1 mRNA expression were measured by qRT-PCR at the indicated time points in 786-O cells after 4 Gy X-ray irradiation. U6 and GAPDH were used as internal controls. **B.** Bod1 protein levels in 786-O cells at indicated time points after 4 Gy X-ray irradiation were measured by Western blot assay. **C.** Relative Bod1 protein levels were quantified using Image J software. Each data point represents the mean of three separate experiments; bars indicate standard errors. *P < 0.05. **P < 0.01.

Because Bod1 depletion causes premature chromatid separation [10], we investigated whether Bod1 was involved in cellular radiation response. The online bioinformatics databases Target Scan (http://www.targetscan.org/) and microRNA.org (http://www.microrna.org/) predicted that Bod1 is a potential target of miR-142-3p. To identify whether both miR-142-3p and Bod1 were involved in the biological effects of irradiation, we measured mature miR-142-3p and Bod1 expression in 786-O cells exposed to X-rays using quantitative RT-PCR (qRT-PCR). As shown in Figure 2A, miR-142-3p expression increased 1 h after irradiation, reached a peak at 4 h, decreased at 8 h, and returned to baseline at 48 h. Meanwhile, Bod1 mRNA expression decreased from 1 h to 4 h after irradiation and then gradually returned to baseline. We then examined Bod1 protein levels in cells after irradiation in a western blot assay. Bod1 protein levels decreased from 1 h to 4 h after exposure to 4 Gy X-rays but increased at the 8 h and 12 h time points (Figure 2B, 2C). These results suggest that radiation affects both miR-142-3p and Bod1 expression, and that miR-142-3p also regulates Bod1 expression.

### MiR-142-3p targets the Bod1 3′-UTR sequence and suppresses its expression

**Figure 3:**
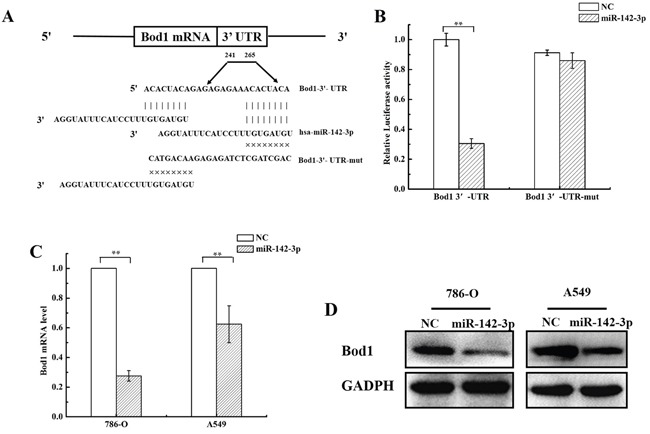
MiR-142-3p targets the Bod1 3′-UTR and suppresses its expression **A.** The putative miR-142-3p binding site in the human Bod1 3′-UTR is shown at the top. The mature miR-142-3p sequence is shown aligned to the target site, and the mutated miR-142-3p seed-pairing sequence is shown below. **B.** Luciferase reporter assays were performed 24 h after 786-O cells were co-transfected with Wt Bod1 or Mut Bod1 vectors together with miR-142-3p mimics or nonsense small RNA oligonucleotides as the negative control. **C.** Relative Bod1 mRNA expression was measured by qRT-PCR after 786-O and A549 cells were transfected with miR-142-3p or negative control (NC). U6 and GAPDH were used as internal controls. **D.** Bod1 protein levels were measured in 786-O in A549 cells by western blot after transfection with miR-142-3p or negative control (NC). Each data point represents the mean of three separate experiments; bars indicate standard errors. **P < 0.01.

Using the Target Scan and microRNA.org databases, we identified two predicted, highly-conserved putative binding sites for miR-142-3p in the 3′-UTR of Bod1 (Figure 3A). To directly investigate interactions between Bod1 and miR-142-3p, we inserted Bod1 3′-UTRs that contained the putative miR-142-3p binding site and Bod1 3′-UTR-mut oligonucleotide pairs with a mutated miR-142-3p binding site into dual-luciferase reporter vectors.

Cells were co-transfected with miR-142-3p mimics and dual-luciferase reporter vector to confirm the target prediction results. Bod1 wild-type 3′-UTR luciferase activity, but not Bod1 3′-UTR-mut activity, decreased in 786-O cells after miR-142-3p transfection (Figure 3B). qRT-PCR and western blot confirmed that Bod1 mRNA and protein levels decreased 48 h after transfection with miR-142-3p in 786-O cells and A549 cells (Figure 3C and 3D). These results indicate that miR-142-3p directly inhibits Bod1 expression by targeting the 3′-UTR of Bod1 mRNA.

### siRNA-induced Bod1 knockdown or miR-142-3p overexpression induce baseline and X-ray-induced premature chromatid separation in 786-O cells

**Figure 4:**
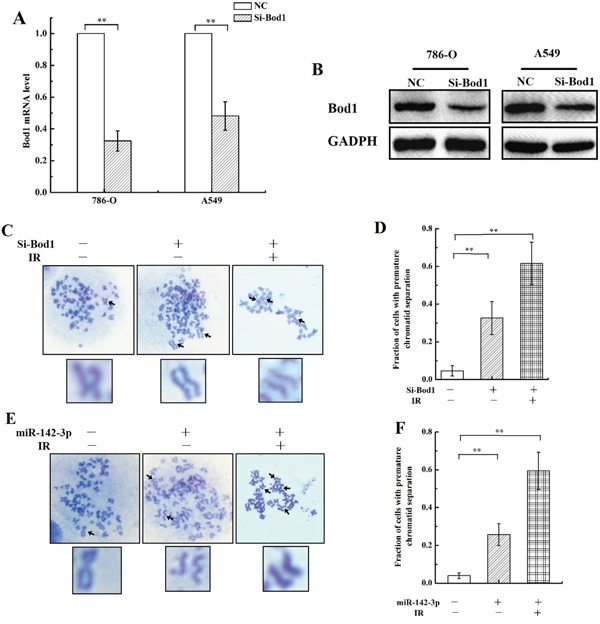
siRNA-induced Bod1 knockdown or miR-142-3p overexpression increases premature chromatid separation in 786-O cells **A.** Relative Bod1 mRNA expression was measured by qRT-PCR in 786-O and A549 cells after transfection with Bod1-siRNA or negative control (NC). GAPDH was used as the internal control. **B.** Bod1 protein level were measured by western blot in 786-O and A549 cells after transfection with Bod1-siRNA or negative control (NC). **C** & **D.** Proportions of 786-O cells with premature chromatid separation in negative control, Bod1 inhibition, and Bod1 inhibition plus irradiation groups based on chromosome configuration analysis. **E** & **F.** Proportions of 786-O cells with premature chromatid separation in negative control, miR-142-3p overexpression, and miR-142-3p overexpression plus irradiation groups based on chromosome configuration analysis. Arrows in the blown-up images indicate a normal chromosome in a negative control cell and chromatids in cells from the two treatment groups. Each data point represents the mean of three separate experiment; bars indicate standard errors. **P < 0.01.

Next, we investigated whether Bod1 and miR-142-3p act together to influence chromatid separation in 786-O and A549 cells. First, we knocked down Bod1 expression in 786-O and A549 cells using siRNA. As shown in Figure 4A and 4B, Bod1 mRNA and protein levels decreased 48 h after siRNA transfection compared to negative control siRNA transfection. Because Bod1 depletion reduces sister chromatid cohesion and increases premature chromatid separation [10], we next measured premature chromatid separation rates in irradiated Bod1 knockdown and negative control 786-O cells. As shown in Figure 4C and 4D, siRNA-induced Bod1 knockdown increased premature chromatid separation compared to negative control treatment, and irradiation further increased premature chromatid separation in Bod1 knockdown cells. We also examined premature chromatid separation in 786-O cells transfected with miR-142-3p mimics. As shown in Figure 4E and 4F, miR-142-3p overexpression also increased premature chromatid separation, and irradiation enhanced this effect.

### Bod1 knockdown or miR-142-3p overexpression increase G2/M phase arrest in 786-O cells

**Figure 5:**
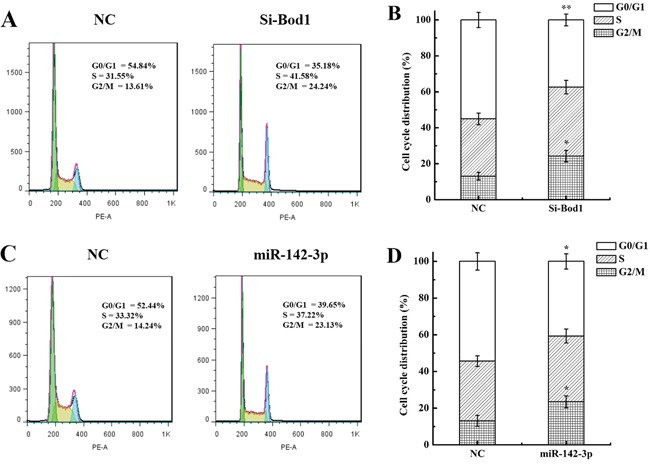
Bod1 knockdown or miR-142-3p overexpression increases cell cycle arrest in 786-O cells **A** & **B.** Cell cycle distributions for Bod1 inhibition or negative control (NC) 786-O cells. **C** & **D.** Cell cycle distributions for miR-142-3p overexpression or negative control (NC) 786-O cells. Each data point represents the mean of three separate experiments; bars indicate standard errors. *P < 0.05; **P < 0.01.

Bod1, which corrects aberrant syntelic attachments in mitotic spindles via activation of the protein phosphatase 2A (PP2A)/Polo-like Kinase 1 (Plk1) pathway [9, 10], is required for proper chromosome biorientation. Because PP2A inhibition increases G2/M phase arrest [24], we investigated whether Bod1 knockdown affected cell cycle progression. Flow cytometry analysis revealed that siRNA-induced Bod1 knockdown increased G2/M phase arrest in 786-O cells compared to negative controls 48 h after transfection (Figure 5A and 5B). Cao *et al.* reported that miR-142-3p also inhibits cell cycle progression and induces G2/M arrest [22]. As shown in Figure 5C and 5D, compared with the control group, G2/M phase arrest increased markedly in miR-142-3p mimics-transfected 786-O cells compared to the control group. miR-142-3p may therefore act through Bod1 to influence cell cycle progression.

### Bod1 knockdown sensitizes 786-O and A549 cells to X-rays

**Figure 6:**
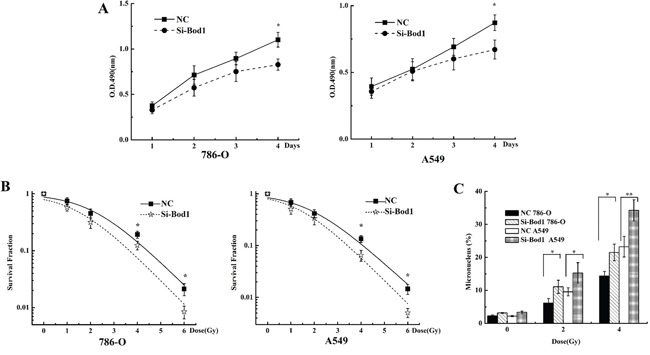
Bod1 knockdown sensitizes 786-O and A549 cells to X-rays **A.** Cell proliferation in 786-O and A549 cells 1, 2, 3, and 4 days after transfection with Bod1-siRNA or negative control (NC) and exposure to 4 Gy X-rays was measured by MTT assay. **B.** Survival in 786-O and A549 cells transfected with Bod1-siRNA or negative control (NC) and then exposed to 0, 1, 2, 4, or 6 Gy X-rays measured by colony formation assay. **C.** Micronucleus counts in 786-O and A549 cells transfected with Bod1-siRNA or negative control (NC) and then exposed to 2 or 4 Gy X-rays. Each data point represents the mean of three separate experiments; bars indicate standard errors. *P < 0.05; **P < 0.01.

We next investigated whether the sensitivity of 786-O and A549 cells to X-rays was affected by siRNA-induced Bod1 knockdown. As shown in Figure 6A, proliferation decreased in 786-O and A549 cells transfected with Bod1-siRNA compared to cells transfected with negative control, especially at 96 h after radiation. Survival also decreased in 786-O and A549 cells transfected with Bod1-siRNA compared to cells transfected with negative control, suggesting that Bod1 knockdown increases the sensitivity of 786-O and A549 cells to radiation (Figure 6B). Bod1 knockdown also increased micronucleus counts compared to negative control-transfected cells after radiation (Figure 6C). Overall, these results suggest that Bod1 knockdown sensitizes both 786-O and A549 cells to X-rays.

### Overexpression of miR-142-3p sensitizes 786-O and A549 cells to X-rays

**Figure 7:**
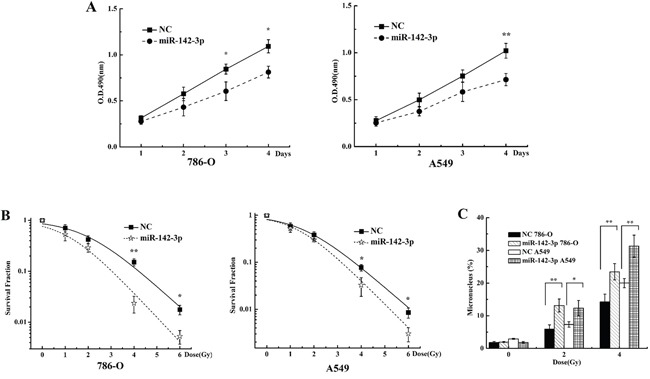
miR-142-3p overexpression sensitizes 786-O and A549 cells to X-rays **A.** Cell proliferation in 786-O and A549 cells 1, 2, 3, or 4 days after transfection with miR-142-3p mimics or negative control (NC) and exposure to 4 Gy X-rays was measured by MTT assay. **B.** Survival in 786-O and A549 cells transfected with miR-142-3p mimics or negative control (NC) and then exposed to 0, 1, 2, 4, or 6 Gy X-rays measured by colony formation assay. **C.** Micronucleus counts in 786-O and A549 cells transfected with miR-142-3p or negative control (NC) and then exposed to 2 or 4 Gy X-rays. Each data point represents the mean of three separate experiments; bars indicate standard errors. *P < 0.05; **P < 0.01.

Because miR-142-3p targeted Bod1, we investigated whether transfection of miR142-3p mimics altered responses to radiation similarly to Bod1 knockdown in 786-O and A549 cells. As shown in Figure 7A, proliferation decreased in 786-O and A549 cells transfected with miR-142-3p mimics compared to negative control-transfected cells, especially at 96 h after radiation. Survival also decreased after radiation in cells transfected with miR-142-3p mimics compared to negative control-transfected cells, especially after the 6 Gy dose (Figure 7B). Additionally, miR-142-3p overexpression increased micronucleus counts after radiation compared to negative control-transfected cells (Figure 7C). Overall, these results suggest that miR-142-3p overexpression also sensitizes 786-O and A549 cells to X-rays by targeting Bod1.

### Bod1 overexpression inhibits miR-142-3p-induced premature chromatid separation

**Figure 8:**
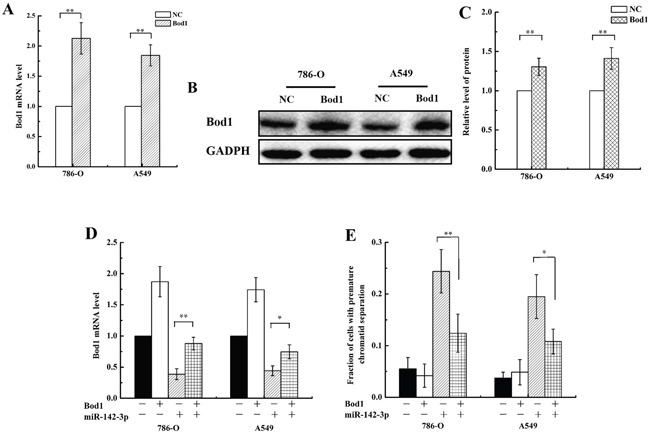
Bod1 overexpression inhibits miR-142-3p-induced premature chromatid separation **A.** Relative Bod1 mRNA expression was measured by qRT-PCR in 786-O and A549 cells after transfection with Bod1 overexpression vector or negative vector (NC). GAPDH was used as the internal control. **B.** Western blot assay of Bod1 protein level were measured in 786-O and A549 cells after transfection with Bod1 overexpression vector or negative vector (NC). **C.** Relative Bod1 protein levels were quantified using Image J software. **D.** Relative Bod1 mRNA expression was measured by qRT-PCR in 786-O and A549 cells after transfection with Bod1 overexpression vector, negative vector, or miR-142-3p and negative mimics. **E.** Proportion of 786-O and A549 cells with premature chromatid separation after transfection of Bod1 overexpression vector, negative vector, or miR-142-3p and negative mimics. Each data point represents the mean of three separate experiments; bars indicate standard errors. *P < 0.05; **P < 0.01.

To determine whether Bod1 overexpression inhibited miR-142-3p-induced premature chromatid separation, we transfected a Bod1 overexpression vector into 786-O and A549 cells. As shown in Figure 8, transfection increased Bod1 mRNA expression (Figure 8A) and protein levels (Figure 8B, 8C) after 48 h compared to negative vector-transfected cells, confirming the efficacy of the Bod1 overexpression construct. Bod1 mRNA expression partially recovered after co-transfection of the Bod1 overexpression vector and miR-142-3p mimics (Figure 8D), suggesting that miR-142-3p overexpression inhibits Bod1 expression. In addition, Bod1 overexpression inhibited miR-142-3p-induced premature chromatid separation compared to negative control cells (Figure 8E).

### Bod1 overexpression inhibits X-ray-induced premature chromatid separation and increases radiation resistance in 786-O and A549 cells

**Figure 9:**
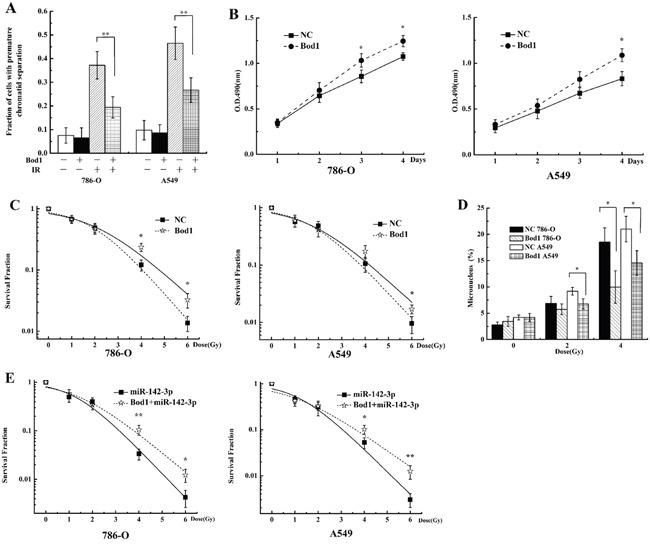
Bod1 overexpression inhibits X-ray-induced premature chromatid separation and enhances resistance to radiation in 786-O and A549 cells **A.** Proportion of 786-O and A549 cells with premature chromatid separation after transfection with Bod1 overexpression vector or negative vector and exposure to 4 Gy X-rays. **B.** Cell proliferation in 786-O and A549 cells 1, 2, 3, or 4 days after transfection with Bod1 overexpression vector or negative vector and exposure to 4 Gy X-rays were measured by MTT assay. **C.** Survival in 786-O and A549 cells transfected with Bod1 overexpression vector or negative vector and then exposed to 0, 1, 2, 4, or 6 Gy X-rays measured by colony formation assay. **D.** Micronucleus counts in 786-O and A549 cells transfected with Bod1 overexpression vector or negative vector and exposed to 2 or 4 Gy X-rays. **E.** Survival in 786-O and A549 cells co-transfected with miR-142-3p and Bod1 overexpression vector and then exposed to 0, 1, 2, 4, or 6 Gy X-rays measured by colony formation assay. Each data point represents the mean of three separate experiments; bars indicate standard errors. *P < 0.05; **P < 0.01.

Because Bod1 knockdown induced premature chromatid separation and sensitized carcinoma cells to X-rays, we investigated whether Bod1 overexpression also affected irradiation-induced premature chromatid separation and radiosensitivity. 786-O and A549 cells were transfected with Bod1 overexpression vector to increase Bod1 levels. Bod1 overexpression inhibited X-ray-induced premature chromatid separation both in 786-O and A549 cells (Figure 9A). Proliferation increased after radiation in 786-O and A549 cells transfected with Bod1 overexpression vector compared to negative vector-transfected cells, especially after 96 h (Figure 9B). Survival also increased after radiation in cells transfected with Bod1 overexpression vector compared to cells transfected with negative vector; this increase was strongest at the 4 and 6 Gy doses (Figure 9C). In addition, miR-142-3p overexpression increased micronucleus counts compared to negative control transfection (Figure 9D). Finally, co-transfection of Bod1 overexpression vector and negative mimics increased survival after radiation compared to co-transfection with negative vector and miR-142-3p (Figure 9E). These results suggest that Bod1 overexpression inhibits X-ray-induced premature chromatid separation and enhances radiation resistance in 786-O and A549 cells.

## DISCUSSION

Proper timing of separation is crucial for balanced chromosome segregation. A cohesin complex maintains connections between sister chromatids until anaphase begins [25–27]. Impaired centromeric cohesion results in premature separation of sister chromatids before anaphase and is associated with aneuploidy [25, 28]. Mutations in genes essential for chromatid cohesion, including Bod1, budding uninhibited by benzimidazoles 1 homolog (Bub1), establishment of cohesion 1 homolog 2 (ESCO2), and nipped-b homolog (NIPBL)/Adherin, all of which are involved in the spindle assembly checkpoint [9, 29–32], result in increased premature chromatid separation.

We found that radiation increased premature chromatid separation in 786-O and A549 cells compared to un-irradiated cells, and western blots and chromosome spread scores confirmed that Bod1 was involved in this effect (Figure 2). Porter *et al.* reported that Bod1 is necessary for the regulation of PP2A activity and Plk1 at the kinetochore and for establishing and maintaining chromatid cohesion [10]. Inhibition of PP2A has antitumor effects in different human cancer cell types [33, 34] and sensitizes nasopharyngeal carcinoma xenografts to radiation by dysregulating mitosis and blocking DNA damage repair [35]. PP2A inhibition-induced sensitization to radiation and chemotherapy in cancer cells is believed to occur via several mechanisms, including sustained phosphorylation of Akt, MDM2, Plk1, TCTP and Cdk1, which are involved in apoptosis, cell cycle deregulation, and inhibition of DNA repair [36–38]. Lv *et al.* reported that inhibition of PP2A increases intracellular p-Plk1, TCTP, and Cdk1 levels and decreases p53 levels, ultimately increasing cell cycle arrest, aberrant mitosis, and radiation-induced inhibition of cell proliferation [24]. These studies together with our results suggest that radiation decreases Bod1 expression and in turn suppress the PP2A/Plk1 pathway. Premature chromatid separation may cause cell cycle arrest and other phenomena involving the miR-142-3p/Bod1/PP2A/PLK1 pathway; future experiments are needed to investigate this possibility.

Epigenetic modifications are important in cancer development and radiation response, and continued investigations of epigenetic mechanisms will likely identify new potential targets for clinical cancer therapies [39]. MiRNAs are involved in the response of tumor cells to radiation and may prove useful as therapeutic agents for enhancing cellular radiosensitivity [40–42]. MiRNAs influence radiation sensitivity in tumors by regulating DNA damage repair, radiation-related signal transduction pathways, the tumor microenvironment, and apoptosis [43, 44]. MiRNAs might also contribute to radiation response by regulating Bod1 expression. Online bioinformatics databases predicted that human miR-142-3p targets the Bod1 gene (Figure 3A). Recently, Chapnik *et al.* reported that miR-142-3p suppresses a group of cytoskeletal regulatory genes, including Bod1, during megakaryopoiesis [45], and miR-142-3p overexpression results in cell cycle arrest in the G2/M phase [22]. We found that miR-142-3p inhibited Bod1 expression (Figure 3B, 3C and 3D) and induced premature chromatid separation (Figure 4B). Furthermore, Bod1 knockdown also increased G2/M phase arrest (Figure 5).

Renal cell carcinoma, a lethal urologic malignancy that originates in renal tubular epithelial cells, comprises approximately 90% of all kidney cancers and accounts for 2-3% of all cancers worldwide [46, 47]; lung cancer is the second most common type of cancer worldwide, with approximately 1.6 million new cases diagnosed each year, and the leading cause of cancer-related death, estimated at 1.37 million annually [48, 49]. Radiotherapy is a crucial component of treatment for these and most other types of cancer, and treatments that increase radiosensitivity in resistant tumor cells would help improve the efficacy of radiotherapy [50, 51]. Because 786-O and A549 cells are resistant to radiation [52, 53], research employing these two cell lines may be particularly helpful in identifying the molecular mechanisms underlying response to radiation and in developing more effective radiotherapies.

miRNAs increase the sensitivity of tumors to radiotherapy by increasing cell cycle arrest [51, 54], and genes that regulate cell cycle progression are important for sensitization to radiation [55–58]. In addition, irradiation alters miRNA expression, suggesting that miRNAs play important roles in irradiation-induced DNA damage and repair [59]. MiR-142-3p specifically might increase radiosensitivity in cells by modulating the expression of its target genes. Our results show that miR-142-3p induced premature chromatid separation by suppressing Bod1 expression, and thus increased sensitivity of 786-O and A549 cells to radiation (Figures 6 and 7). Bod1 overexpression inhibited both radiation- and miR-142-3p-induced premature chromatid separation, which increased resistance to radiation in 786-O and A549 cells. These results might aid in the development of novel therapeutic strategies that use both ionizing radiation and drugs targeting epigenetic mechanisms in carcinoma patients.

In summary, we found that radiation induces premature chromatid separation via the miR-142-3p/Bod1 signaling pathway in 786-O and A549 cells; this mechanism, which involves interactions between epigenetic regulation and radiation response, may be an early contributor to the development of RIGI. Furthermore, we found that irradiation altered miR-142-3p/Bod1 signaling pathway activity, and either knockdown of Bod1 or overexpression of miR-142-3p sensitized 786-O and A549 cells to irradiation. Although the molecular mechanisms underlying premature chromatid separation are not fully understood, our results suggest that epigenetic modifications contribute to early RIGI processes.

## MATERIALS AND METHODS

### Cell culture

786-O cells (human renal carcinoma cells) and A549 cells (human lung carcinoma cells) were obtained from the American Type Culture Collection (Manassas, VA, USA). 786-O and A549 cells were maintained in RPMI-1640 medium (Gibco, USA) supplemented with 10% (v/v) fetal bovine serum (Hyclone, USA) and 1% penicillin/streptomycin (Amresco, USA). Cells were cultured at 37°C in a humidified atmosphere containing 5% CO_2_.

### Radiation

X-ray irradiation was conducted at a Faxitron RX-650 facility (Faxitron Bioptics, USA) at 100 kVp and 5 mA at room temperature. The target of this instrument is wolframium (W), and the dose was 0.97 Gy/min.

### Chromosome spreads

Chromosome spreads were performed as previously described [60]. Briefly, 24 h after transfection or irradiation, cells were incubated with 100 ng/mL colchicine for 4 h. Following a gentle mitotic shake-off, cells were trypsinized, washed with PBS buffer, incubated in 10 mL KCl (0.075 M) at 37°C for 10 min, centrifuged, and fixed in 10 mL fresh Carnoy's fluid (3:1 methanol:acetic acid) for 30 min. The fixed cells were dropped onto 37°C preheated coverslips and dried until grainy. Chromosomal spreads were stained using Giemsa (Solarbio, China). Analyses were performed with an optical microscope (Olympus, Japan) at 100× magnification. At least 50 cell spreads were scored for each sample. The experiment was repeated at least three times.

### Plasmid construction

The 3′-UTR of Bod1 containing the putative miR-142-3p binding site was PCR-amplified from normal human cDNA, and the mutant construct was generated by mutating the miR-142-3p seed sequence (from ACACUACA to CATGACAA and CGATCGAC). The wild-type and mutant Bod1 3′-UTRs were cloned into the psiCHECK2 luciferase vector (Promega, USA). The constructs were verified by DNA sequencing.

### Cell transfection

MiRNA-142-3p mimics and negative controls were purchased from RiboBio (Guangzhou, China). SiRNA targeting Bod1 and negative controls were purchased from GenePharma (Shanghai, China). Bod1 siRNA (5′-GCCACAAAUAGAACGAGCAAUUCAU-3′) was constructed as described [10]. The Bod1 overexpression vector and the negative control vector were purchased from GeneCopoeia (Guangzhou, China). 786-O and A549 cells were plated on the day before transfection at a confluence of 30%−50%, and transfection was performed with Lipofectamine 2000 (Invitrogen, USA) according to the manufacturer's instructions. The medium was exchanged for new culture medium 6 h post-transfection. The cells used in the following experiments were transfected for 48 h.

### Dual-luciferase reporter assay

The Bod1 3′-UTR segments containing the predicted miR-142-3p binding site or a mutated miR-142-3p target site were chemically synthesized by Sangon Biotech (Shanghai, China). These segments were annealed and inserted into the pmirGLO Vector, a dual-luciferase miRNA target expression reporter vector (Promega). 786-O cells were then co-transfected with 150 ng reporter vector and 50 nM miR-142-3p mimics using Lipofectamine 2000 in a 96-well white plate (Corning). Firefly and renilla luciferase activity in cell lysates were assayed using the Dual-Glo Luciferase Assay System (Promega) after transfection, and firefly luciferase activity was normalized to renilla luciferase activity.

### Quantitative real-time polymerase chain reaction (qRT-PCR) was used to measure mRNA expression

Total RNA was extracted from cells with TRIzol Reagent (Invitrogen). Reverse transcription was conducted with the Transcriptor First Strand cDNA Synthesis Kit (Roche, Switzerland) and qRT-PCR was conducted using SYBR Green PCR Master (Roche) to measure mRNA expression. Primers for Bod1 and the GAPDH internal control were purchased from GeneCopoeia. For miRNA detection, reverse transcription and qRT-PCR was performed using an ALL-in-one™ miRNA qRT-PCR Detection Kit (GeneCopoeia). Primers for miR-142-3p and the U6 internal control were also purchased from GeneCopoeia. qRT-PCR was performed using a Bio-Rad Chromo4 System RealTime PCR detector (Bio-Rad, USA). All procedures were conducted according to the manufacturers' protocols. Relative fold-change in mRNA expression was calculated using the 2−^ΔΔ^Ct method with the following equation: RQ (Relative Quantitation) = 2^−ΔΔCt^.

### Western blot

Cells were lysed in RIPA buffer (Beyotime, Shanghai, China) with Protease Inhibitor Cocktail Tablets (Roche, Switzerland). Lysate total protein concentrations were determined using a protein assay kit (Bio-Rad, USA). Equal amounts of protein were denatured with loading buffer (Beyotime) at 100°C for 10 min, then loaded onto a 12% SDS-PAGE gel for electrophoresis and transferred to a methanol-activated polyvinylidene fluoride membrane (Millipore, USA). The membrane was blocked in tris-buffered saline (TBS) containing 5% bovine serum albumin (MP Biomedical, USA) for 2 h at room temperature and then incubated with primary antibodies overnight at 4°C. Bod1 (1:1000, ABGENT, China) and GAPDH (1:1000, ZSGB-BIO, Beijing, China) primary antibodies were used. After washing with TBS, the membrane was incubated with the appropriate horseradish peroxidase (HRP)-labeled secondary antibody for 1 h at room temperature. Secondary antibodies conjugated with HRP included Goat-Anti Rabbit IgG (1:5000, ZSGB-BIO) and Rabbit-Anti Goat IgG (1:5000, ZSGB-BIO). Relative protein levels were quantified using Image J software.

### Flow cytometry analysis

For cell cycle analysis, transfected cells in the logarithmic growth phase were harvested by trypsinization 48 h post-transfection, washed with PBS twice, fixed in 70% ethanol overnight at 4°C, pelleted, resuspended in PBS at 1 × 10^6^ cells/mL, incubated with 100 μg/mL DNase-free RNase A and 0.2% Triton X-100, and stained with 50 μg/mL PI (Sigma, USA) at 4°C for 30 min. A total of 10^4^ nuclei were examined with a BD LSRFortessa™ cell analyzer (BD Biosciences) and DNA histograms were analyzed using Flowjo software. The experiment was repeated at least three times.

### Cell proliferation assay

Cell proliferation was measured using a 3-(4, 5-dimethylthiazol-2-yl)-2, 5-diphenyltetrazolium bromide (MTT) assay. Cells were seeded in 35-mm dishes 24 h before transfection with miRNA-142-3p, siBod1, or both Bod1 vector and miRNA-142-3p. The transfected cells were trypsinized and plated in a 96-well microplate at a density of 2×10^3^ cells/well 24 h after transfection. 24 h later, cells were exposed to X-rays at a dose of 4 Gy. MTT solution (0.5 mg/mL) was added to each well at different time points (1, 2, 3, 4 d) after radiation. After incubation at 37°C for 4 h, cell-free supernatant was removed and the resulting formazan crystals were dissolved in 200 μL of DMSO. Absorbance was measured at 490 nm with a microplate reader. All experimental treatments were repeated in five wells each. The experiment was repeated at least three times.

### Clonogenic survival assay

After irradiation, cells were washed with PBS buffer, trypsinized, and counted using a cell counter (Coulter). An appropriate number of cells were plated into 60-mm dishes to produce colonies. After incubating for 10 d, the cells were fixed with 10 mL fresh Carnoy's fluid and stained with 0.5% crystal violet for 20 min. Colonies with more than 50 remaining cells were counted as survivors. Plating efficiencies (PE) were calculated as follows: number of colonies formed/number of cells plated. Survival ratios were calculated as follows: PE (irradiated)/PE (un-irradiated). All experiments were performed in triplicate and repeated at least three times.

### Micronucleus assay

48 h after radiation, cells were fixed with Carnoy's fluid for 20 min at room temperature and stained with 20 μL of Acridine Orange in an aqueous solution (10 μg/mL). Analyses were performed with a fluorescence microscope (Axio Imager Z2) at 20× magnification. At least 500 cells were scored for each sample. The experiment was repeated at least three times.

### Statistics

Statistical significance (*P* values) of differences in means between two samples were evaluated using Student's *t*-tests. A *P* value < 0.05 (*) was considered statistically significant (**P < 0.01). Graphs show the means ± standard error of at least three independent experiments.
